# Towards scalable production of a collagen-like protein from *Streptococcus pyogenes* for biomedical applications

**DOI:** 10.1186/1475-2859-11-146

**Published:** 2012-11-05

**Authors:** Yong Y Peng, Linda Howell, Violet Stoichevska, Jerome A Werkmeister, Geoff J Dumsday, John A M Ramshaw

**Affiliations:** 1 CSIRO Materials Science and Engineering, Bayview Avenue, Clayton, VIC 3168, Australia

**Keywords:** Bacterial collagen, Recombinant protein production, Bioreactor, High cell density, pCold vector, Defined medium, Fed-batch process

## Abstract

**Background:**

Collagen has proved valuable as biomedical materials for a range of clinical applications, particularly in wound healing. It is normally produced from animal sources, such as from bovines, but concerns have emerged over transmission of diseases. Recombinant collagens would be preferable, but are difficult to produce. Recently, studies have shown that ‘collagens’ from bacteria, including *Streptococcus pyogenes*, can be produced in the laboratory as recombinant products, and that these are biocompatible. In the present study we have established that examples of bacterial collagens can be produced in a bioreactor with high yields providing proof of manufacture of this important group of proteins.

**Results:**

Production trials in shake flask cultures gave low yields of recombinant product, < 1 g/L. Increased yields, of around 1 g/L, were obtained when the shake flask process was transferred to a stirred tank bioreactor, and the yield was further enhanced to around 10 g/L by implementation of a high cell density fed-batch process and the use of suitably formulated fully defined media. Similar yields were obtained with 2 different constructs, one containing an introduced heparin binding domain. The best yields, of up to 19 g/L were obtained using this high cell density strategy, with an extended 24 h production time.

**Conclusions:**

These data have shown that recombinant bacterial collagen from *S. pyogenes*, can be produced in sufficient yield by a scalable microbial production process to give commercially acceptable yields for broad use in biomedical applications.

## Background

Collagens are the major structural proteins in the extracellular matrix of animals, including all vertebrates and invertebrates [1,2]. Collagens are defined by a characteristic triple-helix structure in which 3 polyproline-II-like helices are super-coiled around a common axis to give a rope-like structure [3]. In this structure, the only amino acid that is small enough to fit within the centre of the triple-helix is glycine (Gly), leading to a characteristic, repeating amino acid sequence for the collagen triple-helix of (Gly-Xaa-Yaa)_n_ , where Xaa and Yaa can be any amino acid, but are frequently proline (Pro) as Xaa and 4-hydroxyproline (Hyp) as Yaa [3]. Only a limited number of Gly-Xaa-Yaa triplet sequences are found in high frequency, while over one third of all possible triplets have not been observed in natural sequences [4]. Those that occur in higher frequency are often associated with sequences that enhance the stability of the triple helix, and include segments where hydrophobic residues or charged pair interactions are present, as well as Hyp containing sequences [5].

Collagen has been proven safe and effective in a wide variety of medical products and in various clinical applications [6,7]. For medical purposes, collagen is usually extracted from animal sources, especially bovine, but there has been a growing concern of transmissible diseases arising from use of this source. The ready availability of a cost effective recombinant collagen would, therefore, be a clear advantage. Progress has been made in developing the commercial production of human recombinant collagens in yeasts [8-11], with yields of around 2 g/L being achieved in *Pichia pastoris*[12]. However, this system requires the co-expression of the enzyme prolyl 4-hydroxylase (P4H) which leads to secondary modification of certain Pro residues to give Hyp that contributes to collagen stability. This, in turn, requires additional oxygenation during the fermentation process to facilitate hydroxylation as well as the addition of methanol for induction, adding to the complexity and cost of production [12]. Previously, the incorporation of Hyp in *Escherichia coli* cultures had been proposed, through incorporation of Hyp in the media [13], but this leads to non-specific incorporation into both the Xaa and Yaa positions in the triple helix. More recently, specific incorporation of Hyp into the Yaa position in *E. coli* cultures, but for only small collagen peptides, has been reported using a biosynthetic shunt to produce ascorbate-like entities that drive heterologously expressed P4H [14].

Collagen-like triple-helical sequences with Gly at every third residue and a high Pro content have been observed in various bacteria [15-17]. These bacterial collagen-like proteins lack prolyl hydroxylation, yet several have been shown to form stable triple-helices at 35–38°C [17], through use of alternative stabilisation motifs [18]. These collagen proteins can, therefore, be expressed as recombinant products in an *E. coli* system without the need for P4H co-expression [15,18].

The bacterial collagen examined in this present study, with its limited biological interactions behaves like a ‘blank slate’, which could be used in clinical applications such as wound management or adhesion prevention. It can be modified to include additional copies of the collagen domain [19] or by inclusion of other mammalian collagen sequences [20] or other triple helical proteins. For example, it is possible to introduce specific functions, such as cell binding via a mammalian integrin binding site [21,22] or inclusion of a heparin binding domain [23,24] into the bacterial collagen domain.

In this study we have produced the recombinant collagen-like protein Scl2 from *S. pyogenes* in *E. coli* using a stirred tank bioreactor. The Scl2 sequence, including the registration domain (V-domain), the collagen domain (CL), but without the C-terminal non-collagen tail, was inserted into a pColdIII (Takara Bio Inc.) vector for expression in *E. coli*[18]. As an example of this new class of collagens, the recombinant Scl2 gene construct from *S. pyogenes* has previously been expressed in shake flasks [18,25]. The purified collagen protein was shown to be non-cytotoxic and non-immunogenic, while supporting cell attachment, although cell spreading was limited at 16 h [25].

If this bacterial collagen or its modified sequences are to be used as new clinical materials, it is important that they can be produced in commercially feasible quantities at a competitive cost for biomedical product manufacture. This study has established that this collagen can be produced in good yield in a bioreactor using a cost effective fully defined medium.

## Results and discussion

### Protein expression constructs

**Figure 1:**
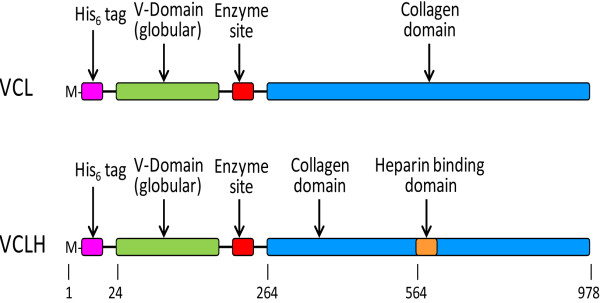
**Schematic diagrams showing the VCL and VCLH constructs.** Base pair numbers are indicated below the schematics. The His-Tag and Enzyme Site domains have been added to the naturally occurring *S. pyogenes* Scl2.28 gene [15] as previously described [25].

**Figure 2:**
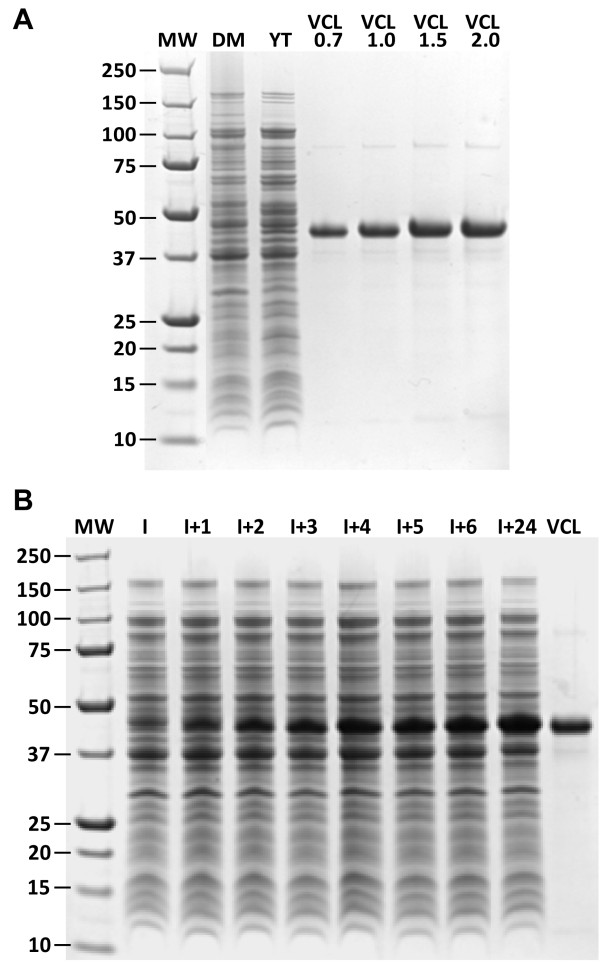
**SDS-PAGE analysis of protein expression.(A)** Protein expression in shake flask cultures. MW, molecular weight standards; DM, defined medium; YT, complex medium (2xYT) and purified VCL protein standards 0.7 to 2.0 μg. **(B)** Protein expression time course, fed-batch process with stepwise temperature reduction after induction. MW, molecular weight standards; I, pre-induction; I + 1 to I + 24 hours after induction; VCL, purified VCL standard.

In the present study, recombinant bacterial collagen, VCL, has been expressed using the pCold vector system, as it had previously been shown that this system was effective in shaker culture for production of the protein in the *E .coli* host strain BL21 [18,25]. The construct was termed VCL, where V is the non-triple-helical registration domain and CL is the triple-helical, collagen-like domain (Figure 1). The pCold vectors selectively induce target protein synthesis at low temperatures where the synthesis of host proteins is reduced or suppressed and protease activity is decreased. The pCold system is designed for efficient protein expression utilising a promoter derived from the *cspA* gene, which is a cold-shock gene, with a *lac* operator inserted downstream to control expression [26]. In addition, expression of a second construct, VCLH, which included a heparin binding domain, H, substituted using site directed mutagenesis into the CL sequence, was also tested. In this construct, a heparin binding sequence, GRPGKRGKQGQK, derived from the collagenous tail of acetylcholine esterase [23,24] was substituted via PCR directed integration into the CL sequence GPAGPMGPAGER that starts at base pair 564/amino acid residue 188 (Figure 1). The substitution was confirmed by DNA sequencing. The pCold vector system is available as a research tool; it is possible that proprietary commercial vectors could give much better yields than observed in the present study that has used the pCold system. Recombinant proteins, VCL and VCLH, were isolated in sufficient quantities for use as electrophoretic quantitation standards (Figure 2A) using a conventional purification protocol (Ni metal affinity column chromatography and with binding via a His_6_ tag incorporated at the N-terminus of the protein). Final purification was achieved by gel permeation chromatography [25].

### Protein expression in shake flasks

**Figure 3:**
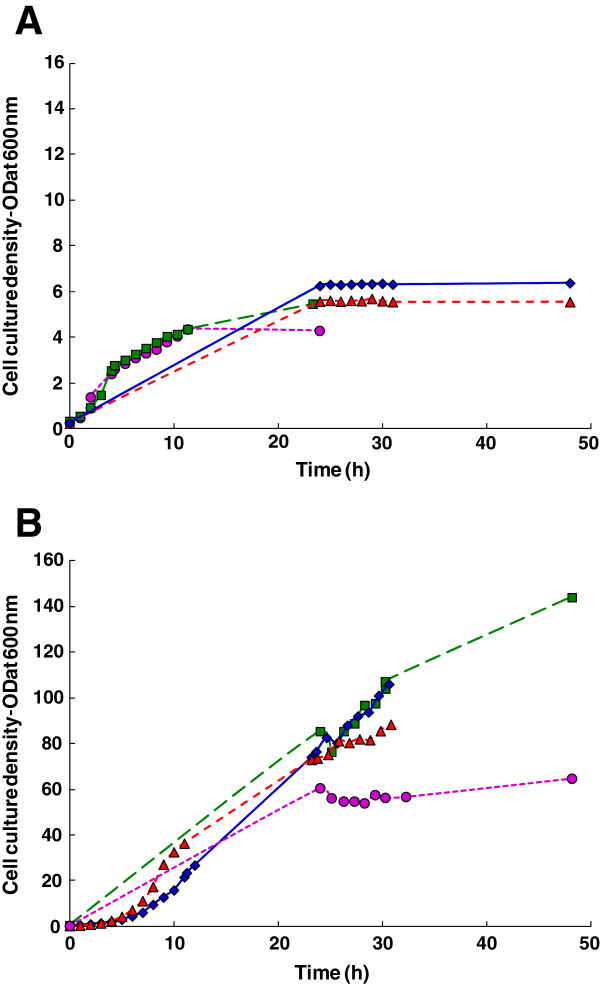
**Effect of fermentation process design and induction temperature on growth of collagen-producing *E. coli *strains.** Cell concentration was estimated by measuring optical density at 600 nm. See Table 1 for experimental details. **(A)** Shake flask and low cell density processes.♦, VCL, shake flask, complex medium (2xYT); ▲, VCL, shake flask, defined medium; ■, VCL, bioreactor batch process (low cell density); ●, VCLH, bioreactor batch process (low cell density). **(B)** High cell density processes. ♦, VCLH, bioreactor fed-batch process, induction at 25°C for 10 hours; ▲, VCL, bioreactor fed-batch process, induction at 25°C for 10 hours; ●, VCL, bioreactor fed-batch process, induction at 15°C for 24 hours; ■, VCL, bioreactor fed-batch process, induction for 24 hours with stepwise temperature reduction.

The expression of VCL in shake flasks using 2xYT Media and Defined Media (DM) were used as the baseline process for this study. Cells were grown for 24 h at 37°C, after which an OD_600 nm_ of around 6 was achieved, prior to induction at 25°C. Expressed protein production was determined after incubation at 25°C for 10 h, followed by a further 14 h at 15°C. It was hoped that the additional reduction in temperature would maintain the selective target protein production achieved by the pCold vector system. The changes in cell densities were tracked throughout the process (Figure 3A), and at the end of the production phase, moderate amounts of wet cell paste, 8.3 g/L for DM and 9.7 g/L for 2xYT, were obtained. Expression yields were determined by comparison to known amounts of VCL protein by SDS-PAGE (Figure 2A). These shake flask processes achieved recombinant collagen expression levels of around 0.2 g/L in DM and 0.3 g/L in 2xYT (Table 1). These yields are adequate for biochemical and biophysical studies of VCL and related constructs such as VCLH, but are well below production levels required for commercial production.

**Table 1:** Fermentation conditions and protein yields for VCL and VCLH constructs

**Construct**	**Process**	**Pre- induction phase**	**Induction temperature (°C)**	**Induction phase**	**Final OD (600 nm)**	**Wet cell paste (g/L)**	**Volumetric productivity (g/L)**	**Specific productivity (g/OD**_**600 nm**_**)**
VCL	Flask Complex medium	24 h at 37°C	25	25°C for 10 h 15°C for 14 h	6.4	9.7	0.3	0.05
VCL	Flask Defined medium	24 h at 37°C	25	25°C for 10 h 15°C for 14 h	5.6	8.3	0.2	0.04
VCL	Batch Defined medium	5 h at 37°C	25	25°C for 7 h	5.5	5.3	0.7	0.13
VCLH	Batch Defined medium	5 h at 37°C	25	25°C for 7 h	4.3	5.8	1.0	0.23
VCLH	Fed batch Defined medium	24 h at 37°C	25	25°C for 10 h	106	108	8.2	0.08
	Fed batch Defined medium	24 h at 37°C	25	25°C for 10 h	88	113	9.5	0.11
VCL	Fed batch Defined medium	24 h at 37°C	15	15°C for 24 h	65	112	10.0	0.15
VCL	Fed batch Defined medium	24 h at 37°C	25	25°C for 10 h 20°C for 5 h 15°C for 9 h	96	n.d.	19.3	0.20
VCL	Fed batch Defined medium	24 h at 37°C	25	25°C for 10 h 20°C for 5 h 15 °C for 9 h	105	137	13.0	0.12
VCL	Fed batch Defined medium	24 h at 37°C	25	25°C for 10 h 20°C for 5 h 15°C for 9 h	144	148	19.0	0.13

### Protein expression in 2 L stirred tanks – batch process (low cell density)

Expression of both the VCL and the VCLH was tested using a batch process in a 2 L stirred tank bioreactor. After inoculation, both processes were maintained at 37°C for the first 5 hours to increase the cell density prior to induction. After this initial period, the culture had an optical density OD_600 nm_ in the range of 2 to 3. The cultures were then cooled to 25°C, induced with IPTG and incubated for a further 7 h. For both constructs, wet cell paste yields were between 5 and 6 g/L. The increase in cell density was tracked throughout (Figure 3A), showing, steady increases in both cases. The final optical densities were below those achieved in shake flasks reflecting the shorter times for both the growth and induction phases in the bioreactor experiments. Despite the shorter incubation times, both trials led to moderate recombinant collagen yields, 0.7 to 1.0 g/L. Transfer of the process to a more controlled bioreactor resulted in a 3-fold increase in total collagen production and a 4-fold increase in specific productivity (mass of collagen per cell), although further yield increases are required to meet the requirements of commercial production.

### Protein expression in 2L stirred tanks – fed-batch process (high cell density)

Both the VCL and VCLH constructs were used for initial testing of expression using a high cell density, fed-batch process. A high cell density process was implemented to increase collagen production. If the specific collagen productivity (mass of collagen per cell) can be maintained then an increase in cell density will result in an increase in collagen production. To achieve a high cell density prior to induction, the initial growth phase was extended to 24 h during which cell densities typically exceeding OD_600 nm_ of 80 were obtained. Again, the temperature was reduced to 25°C and recombinant protein expression induced by the addition of IPTG; the culture was incubated for a further 10 h. The process resulted in a significant improvement in collagen yields; 8.2 g/L for VCLH and 9.5 g/L for VCL, indicating that this fermentation approach has potential for high level production of the recombinant protein. The specific productivity was less compared to the low cell density process but due to the large increase in cell mass the volumetric productivity increased almost 10-fold. As the differences between VCL and VCLH production were relatively small, only VCL was used for further process optimisation.

The previous experiment showed that a 12°C temperature decrease resulted in collagen yields in excess of 8 g/L. The effect of induction temperature was examined further by following a similar protocol, except the induction was changed from 25°C to 15°C and the induction time was increased from 10 to 24 h. This process was used to test whether a lower temperature would further reduce host cell protein production resulting in greater enrichment of recombinant collagen. The lower induction temperature did result in greater enrichment of the target protein (the specific productivity increased 2-fold) whilst volumetric production was similar to the previous experiment (9.5 g/L at 25°C and 10 g/L at 15°C). The final optical density was lower at 15°C (OD_600 nm_= 65) compared to 25°C (OD_600 nm_= 88) despite the induction time being increased from 10 to 24 hours. The unchanged volumetric production was most likely the result of slower cell growth at the lower temperature as shown in Figure 3B; cell growth is negligible after reducing the temperature to 15°C.

To further increase recombinant collagen production the extended induction time was retained and stepwise temperature reduction was introduced into the process to enhance pCold’s “cold shock selectivity”. After induction for 10 h at 25°C, the temperature was reduced to 20°C and the culture held at this temperature for 5 h. The temperature was then reduced again to 15°C and held at this temperature for a further 9 h. This process change resulted in a further marked yield increase with recombinant collagen production levels of 13.0, 19.0 and 19.3 g/L in three separate batches (average of 17.1 g/L) – production levels that are potentially within a commercially feasible range. The stepwise temperature reduction resulted in an increase in final optical density without too greater impact on specific productivity. Recombinant collagen production was followed during the induction phase of the process using SDS-PAGE (Figure 2B) which shows a steady increase in VCL production during the initial hours after induction with a further increase at the end of the process. In addition, purified protein samples from cell aliquots, for use as gel standards, also showed that the collagen domains of the expressed products were resistant to trypsin digestion [19] indicating that the collagen domains had folded into the triple-helical conformation which confers proteolytic resistance [19].

In the present study, we have shown that good production levels of VCL can be achieved, with a maximum production of 19.3 g/L. After removal of the His_6_ tag and the V-domain, which would probably be necessary for biomedical applications of the collagen-like domain, this equates to a maximum yield of the CL domain of about 14.1 g/L, with a further loss of yield depending on the efficiency of any purification protocol. The use of the His_6_ tag is efficient for laboratory purifications [19,25], but would not be suitable for large scale commercial preparations. Other affinity approaches may be more useful and cost effective, including the use of specific collagen binding proteins [27]. The present study has shown that excellent recombinant collagen yields can be obtained using a high cell density fed-batch process using pCold vectors. The best yields obtained compare favourably with the average yields reported for other bacterial expression studies of 14 g/L [28], although there may be higher commercial yields that remain commercial-in-confidence. Nevertheless, use of alternative vector systems that may be better suited for use at high cell densities may lead to even further increases in yield. The present yield, however, compares favourably with fed-batch fermentation yields obtained using *P. pastoris* and *Hansenula polymorpha*. These recombinant systems were used to produce a 30 kDa fragment containing an N-propeptide which after cleavage gives a 14 kDa mammalian collagen fragment in the yeasts *P. pastoris* and *Hansenula polymorpha* (recently designated as a *Pichia* species) [10]. These data gave the best yield with *H. polymorpha* expression of around 0.6 g/L, well below the bacterial collagen yields achieved in this study. Nevertheless, much better yields, better than 2 g/L, could be obtained from *P. pastoris* when used in commercially optimised production [12].

## Conclusions

An initial optimisation approach of the present pCold vector system showed that recombinant bacterial collagen can be produced in large quantities in *E. coli,* with average production levels of 17.1 g/L, much greater than has been achieved for mammalian collagens in yeast expression systems [12]. This was achieved at low cost through use of a fully defined medium, which was formulated without any animal products, and without the introduction of any media components that could have a negative impact on downstream processing. The bacterial production system was not affected by the introduction of a heparin binding sequence, with comparable yields being obtained for both the VCL and VCLH constructs.

Bacterial collagen is an excellent alternative source of collagen for use in biomaterial applications and as a scaffold for tissue engineering. As a bacterially expressed product, it can be made as a non-animal collagen in an animal free system. As an expressed product, it does not have the variation in quality, purity and predictability of performance that can be found for animal derived materials, nor does it carry the risk of transmission of infectious agents. The properties of the bacterial collagen from *S. pyogenes* have been characterised [25]. It has been shown that this bacterial collagen is biocompatible, non-cytotoxic and non-immunogenic [25] and hence suitable for biomaterial applications. This collagen is stable without the need for secondary modifications, especially proline hydroxylation. The bacterial collagen system also allows for the design of novel functional materials, such as the inclusion of selected binding domains, eg: introducing integrin binding domains [22] or heparin binding into the natural bacterial collagen sequence.

## Methods

### Recombinant construct

The fragment of *S. pyogenes* Scl2.28 gene [15] encoding the globular (V) and the collagen-like (CL) domains, and the variations to include a His-tag at the N-terminal of the V-domain and an enzyme cleavage site between the V- and CL domains, was cloned into the pColdIII vector (Takara Bio, Shiga, Japan), giving construct VCL, and transformed for expression in *E. coli* strain BL21 as previously described [25]. In addition, the construct was cloned into the shuttle vector pSL1180 using restriction sites 5^′^ NdeI and 3^′^ BamHI. This clone was used for site directed mutagenesis to introduce a heparin binding sequence [23,24] GRPGKRGKQGQK at base pair 564 via PCR directed integration, giving construct VCLH. PCR products were treated with DpnI, to digest all parental DNA and subsequently transformed into *E. coli* strain XL1-BLUE. Selected DNA clones were sequenced and those containing the required heparin binding domain was subcloned into pColdIII vector (Takara Bio, Shiga, Japan) using the 5^′^ NdeI and 3^′^ BamHI sites. Positive clones were selected and transformed into *E. coli* host BL21 for expression. Schematics of the two constructs are shown in Figure 1. Prior to expression studies, all constructs were confirmed by DNA sequencing.

### Media

Complex medium (2xYT) contained 16 g tryptone, 10 g yeast extract and 5 g NaCl per litre. The defined medium (DM) used in this study contained per litre: KH_2_PO_4_, 10.6 g; (NH_4_)_2_HPO_4_, 4.0 g; citric acid, 1.7 g; glucose, 25 g; MgSO_4_.7H_2_O, 1.23 g; ampicillin, 200 mg; thiamine hydrochloride, 4.4 mg; and trace salts solution 5 mL. The trace salts solution contained per litre: CuSO_4_.5H_2_O, 2.0 g; NaI, 0.08 g; MnSO_4_.H_2_O, 3.0 g; Na_2_MoO_4_.2H_2_O, 0.2 g; boric acid, 0.02 g; CoCl_2_.6H_2_O, 0.5 g; ZnCl_2_, 7.0 g; FeSO_4_.7H_2_O, 22.0 g; CaSO_4_.2H_2_O, 0.5 g and H_2_SO_4_, 1 mL. As required, glucose, magnesium, trace salts, thiamine and ampicillin were aseptically added as concentrated stock solutions to the media after sterilisation.

### Seed cultures

Primary seed cultures were prepared from single colonies taken from a fresh transformation plate, and grown in 10 mL of 2xYT (in a 30 mL bottle) containing 10 g/L glucose and 200 μg/mL ampicillin. The culture was incubated at 37°C shaking at 200 rpm for 24 h. A volume (0.5 mL) of the primary seed culture was used to inoculate 500 mL of 2xYT (in a 2L Erlenmeyer flask) containing 10 g/L glucose and 200 μg/mL ampicillin. This secondary seed culture was incubated at 37°C shaking at 200 rpm for 16 h.

### Protein expression in shake flasks

Cultures were grown in 2 L Erlenmeyer flasks containing 500 mL of either 2xYT or DM media and incubated at 37°C for 24 h. The cultures were then cooled to 25°C and protein expression induced by addition of 0.5 mM isopropyl beta-D-thiogalactopyranoside (IPTG). The cultures were then incubated at 25°C for 10 h and for a further 14 h at 15°C. Samples were taken during the course of the incubation and the cells pelleted by centrifugation followed by disruption using Bugbuster™ (Merck) and quantification by SDS-PAGE (as described below).

### Protein expression in 2L stirred tanks

Recombinant bacterial collagens were produced in 2 L stirred tank bioreactors connected to a Biostat B (Sartorius Stedim Germany) control system. The initial volume of medium in the fermenter was 1.6 L and glucose as used as the carbon source. A volume of the secondary seed culture was added to the bioreactor to attain an initial optical density (measured at 600 nm) of 0.25. Foaming was controlled via the automatic addition of 10% (v/v) polypropylene glycol 2025; 3 mL of the antifoam solution was added prior to inoculation. The pH setpoint was 7.0, controlled by automatic addition of either 10% (v/v) H_3_PO_4_ or 10% (v/v) NH_3_ solutions. The dissolved oxygen setpoint was 20% of saturation and a two-step cascade control was used maintain the dissolved oxygen above the specified setpoint. The agitator speed ranged from 500 rpm to 1200 rpm and airflow (supplemented with 5% pure O_2_) ranged from 0.3 L min^-1^ to 1.5 L min^-1^. For the high cell density fed-batch processes, the feed solution was comprised of 400 mL of 660 g/L glucose solution to which 40 mL of 1 M MgSO_4_7H_2_O added. The feed flow rate was 15 mL h^-1^ and the feed was initiated 8.5 h after inoculation. Incubation times and temperatures for individual experiments are given in Table 1. The culture was cooled (over a 20 minute period) to the required temperature 24 h after inoculation to activate the cold shock component of the vector and protein expression induced by addition of 1 mM (final concentration in the culture) IPTG.

### Preparation of protein standards

Recombinant VCL and VCLH protein standards were extracted from wet cell paste by sonication in 40 mM sodium phosphate buffer, pH8.0, 1 mM phenylmethanesulfonyl fluoride, using 20 ml buffer per gram of cell paste. After centrifugation, clarified supernatant was taken to 20 mM sodium phosphate 300 mM NaCl and 30 mM imidazole buffer, pH8.5, and absorbed onto a Ni charged HyperCel-Sepharose metal ion affinity resin (Pall Life Sciences). Elution was by the same buffer, but containing 500 mM imidazole. Eluted fractions containing recombinant protein were pooled, concentrated and exchanged into 20 mM sodium phosphate buffer, pH8.0, using a 10 kDa cross-flow filtration membrane (Pall Life Sciences), followed by further purification in the same buffer on a Sephacryl S200 26/60 column (GE Healthcare). After additional treatment of purified VCL and VCLH with trypsin [19], SDS-PAGE [29] as described for Sample Analysis, was used to show that the triple helix remained intact.

### Sample analyses

Samples were taken from the cultures throughout each process. Cell mass was determined by measuring the optical density of diluted samples at 600 nm (OD_600 nm_). VCL or VCLH were quantified in small sub-samples and to correct for changes in cell numbers, the formula 200/OD_600 nm_ was used to determine the volume of culture required for SDS PAGE analysis. The aliquot was centrifuged and the cell pellet lysed by addition of 40 μL of Bugbuster™ (Merck) and incubated for 1 h. After the incubation, 10 μL 5x sample buffer containing 5% (v/v) 2-mercaptoethanol was added to the lysate and the mixture heated at 95°C for 1 min prior to electrophoresis. SDS PAGE was completed as described by [29] using NuPAGE (Invitrogen) 4-12% Bis-Tris gels with MES running gel buffer, at 180 V for 60 min, followed by staining with Coomassie Blue R-250. After destaining, the band corresponding to VCL or VCLH bands were quantified by densitometric analysis using Multi Gauge V3.0 FujiFilm software and compared to known standard loadings of purified VCL protein.

## Competing interests

All the authors declare that they have no competing interests.

## Authors’ contributions

YYP carried out construct production, shaker flask production, protein standard purification, yield quantitation and assisted in drafting the manuscript, LH carried out fermentation studies and VS carried out construct production and shaker flask production. JAW supervised the construct production and reviewed and revised the manuscript, GJD supervised design and implementation of the fermentation studies and reviewed and revised the manuscript. JAMR supervised the project and drafted the manuscript. All authors read and approved the final manuscript.
